# Testing Empathy in Machines: A Comparative Study of Large Language Models and Motivational Interviewing

**DOI:** 10.1192/j.eurpsy.2026.12058

**Published:** 2026-07-31

**Authors:** J. B. Rodrigues, J. P. Azenha, P. Trindade, C. Adão

**Affiliations:** Departamento de Psiquiatria e Saúde Mental, Unidade Local de Saúde de Lisboa Ocidental, Lisboa, Portugal

## Abstract

**Introduction:**

Large language models (LLMs) are increasingly proposed as tools for behavioral healthcare, with the potential to augment or even automate aspects of psychotherapy. Among therapeutic frameworks, motivational interviewing (MI) is especially relevant as it is well-validated for eliciting behavior change and improving adherence. MI’s structured, empathetic style provides a rigorous benchmark for assessing whether LLMs can replicate therapeutic dialogue. While LLMs promise scalability and accessibility, their application in psychotherapy remains high-stakes, raising concerns of safety, fidelity, and ethical deployment.

**Objectives:**

This study aims to evaluate whether current leading LLMs reproduce the principles of MI, focusing on empathy, change talk, rolling with resistance, and support for autonomy.

**Methods:**

A standardized MI script addressing ambivalence toward alcohol reduction was applied across five leading LLMs: GPT-4, GPT-3.5, Claude, Gemini, and Llama-2. Outputs were coded against MI core processes (engaging, focusing, evoking, planning), MI principles (empathy, discrepancy, resistance management, non-confrontation, self-efficacy), and common techniques (open questions, reflections, affirmations, eliciting change talk, avoidance of directive advice).

**Results:**

GPT-4 most consistently embodied MI: it engaged and focused effectively, elicited change talk, and demonstrated empathy through accurate reflections. It avoided confrontation and supported self-efficacy, applying all five principles with notable fidelity. Claude showed similar strengths, especially in empathy and discrepancy development, but was less reliable in rolling with resistance. Gemini balanced empathy with structure, producing clear open questions and affirmations, but defaulted to directive recommendations, limiting adherence to non-confrontation and autonomy support. GPT-3.5 generated open questions but frequently lapsed into advice-giving, undermining MI principles and the planning process. Llama-2 showed the lowest fidelity, often offering prescriptive advice rather than reflections, with minimal engagement in evoking change talk or consolidating commitment. Across models, nuanced techniques such as managing resistance and strengthening commitment were inconsistently applied, with GPT-4 and Claude demonstrating the highest overall fidelity to MI principles and techniques.

**Conclusions:**

LLMs can reproduce surface features of MI and show promise for scaling access to supportive dialogue. However, consistent therapeutic stance and deeper MI techniques remain beyond current capacity. These findings echo concerns that while LLMs may simulate skills, they do not yet deliver the clinical sensitivity required for psychotherapy. Responsible development should prioritize domain-specific training, interdisciplinary oversight, and hybrid models where clinician supervision ensures therapeutic integrity.

**Disclosure of Interest:**

None Declared

